# Sequencing patterns of ventilatory indices in less trained adults

**DOI:** 10.3389/fspor.2022.1066131

**Published:** 2023-01-23

**Authors:** Martin Pühringer, Susanne Ring-Dimitriou, Bernhard Iglseder, Vanessa Frey, Eugen Trinka, Bernhard Paulweber

**Affiliations:** ^1^ Department of Sport and Exercise Science, University of Salzburg, Salzburg, Austria; ^2^ Department of Geriatric Medicine, Christian-Doppler-Clinic, Paracelsus Medical University, Salzburg, Austria; ^3^ Department of Neurology, Christian Doppler University Hospital, Paracelsus Medical University and Centre for Cognitive Neuroscience, Affiliated Member of the European Reference Network EpiCARE, Salzburg, Austria; ^4^ Neuroscience Institute, Christian Doppler University Hospital, Paracelsus Medical University and Centre for Cognitive Neuroscience, Salzburg, Austria; ^5^ Department of Internal Medicine I, Paracelsus Medical University, Salzburg, Austria

**Keywords:** exercise testing (CPET), exercise physiology, aerobic capacity, anaerobic threshold (AT), point of optimal ventilatory efficiency (POE), fat oxidation, indirect calorimetry

## Abstract

Submaximal ventilatory indices, i.e., point of optimal ventilatory efficiency (POE) and anaerobic threshold (AT), are valuable indicators to assess the metabolic and ventilatory response during cardiopulmonary exercise testing (CPET). The order in which the ventilatory indices occur (ventilatory indices sequencing pattern, VISP), may yield additional information for the interpretation of CPET results and for exercise intensity prescription. Therefore, we determined whether different VISP groups concerning POE and AT exist. Additionally, we analysed fat metabolism *via* the exercise intensity eliciting the highest fat oxidation rate (Fat_max_) as a possible explanation for differences between VISP groups. 761 less trained adults (41–68 years) completed an incremental exercise test on a cycle ergometer until volitional exhaustion. The ventilatory indices were determined using automatic and visual detection methods, and Fat_max_ was determined using indirect calorimetry. Our study identified two VISP groups with a lower work rate at POE compared to AT in VISP_POE < AT_ but not in group VISP_POE = AT_. Therefore, training prescription based on POE rather than AT would result in different exercise intensity recommendations in 66% of the study participants and consequently in unintended physiological adaptions. VISP_POE < AT_ participants were not different to VISP_POE = AT_ participants concerning VO_2peak_ and Fat_max_. However, participants exhibiting a difference in work rate (VISP_POE < AT_) were characterized by a higher aerobic capacity at submaximal work rate compared to VISP_POE = AT_. Thus, analysing VISP may help to gain new insights into the complex ventilatory and metabolic response to exercise. But a methodological framework still must be established.

## 1. Introduction

Cardiorespiratory fitness is an indicator of physical performance, and cardiovascular and metabolic health. Accordingly, the improvement of the cardiorespiratory fitness is recommended in prevention and therapy to lower the risk of morbidity and mortality (1–3). In cardiopulmonary exercise testing (CPET) two approaches developed over the decades to detect changes in cardiorespiratory fitness: the testing of the aerobic power, i.e., maximal oxygen uptake: VO_2max_, and the aerobic capacity, i.e., percent of VO_2max_ expressed as AT (3, 4). VO_2max_ is the most common applied marker to monitor changes of the exercise performance level and to prescribe a health-enhancing exercise intensity in prevention and therapy. However, submaximal ventilatory indices have been shown to be an alternative basis for exercise intensity prescription and a better predictor of physical performance, morbidity and mortality (1, 2, 5). Different submaximal ventilatory indices (e.g., POE, AT, respiratory compensation point (RCP)) have been established within the last decades in CPET and a time-dependent sequence of these indices associated to the three-phase model of energy supply was reported (2, 3). Although these submaximal ventilatory indices have been investigated extensively, only few studies discussed the time-dependent sequencing patterns of these various ventilatory indices in adults (6–8). It is, therefore, important to analyse differences in VISP to improve the interpretation of CPET data regarding health-enhancing physical exercise prescription.

The determination of ventilatory indices from CPET data has a long tradition (3, 9) and these indices detected from ventilatory variables and measured by indirect calorimetry can be used to non-invasively detect exercise-induced changes of the metabolism (2, 10). Traditionally a three-phase model with two submaximal ventilatory indices, in particular the AT and the RCP, is used to discriminate three phases of energy supply during an incremental CPET (2, 11–13). The first increase in blood lactate concentration during an incremental exercise test leads to a disproportionate increase in carbon dioxide output (VCO_2_) in relation to oxygen uptake (VO_2_) due to the “excess CO_2_” from the bicarbonate buffering of H^+^ resulting from the dissociation of lactic acid. This gas exchange response characteristic is used for the determination of the AT using the v-slope method (3, 14) and marks the transition from a predominantly aerobic (mainly fatty acid oxidation and aerobic glycolysis; phase one) to a partially anaerobic energy metabolism (mainly aerobic and anaerobic glycolysis; phase two). As a consequence to the slightly increased end-tidal carbon dioxide partial pressure (PETCO_2_), it is stated that there is a compensatory stimulus for ventilation mediated *via* the carotid bodies to regulate the lactic acid-induced acidosis (2). Theoretically, this leads to a simultaneous increase in minute ventilation (VE) at the AT (3). Therefore, it is common practice to determine the AT *via* the POE-detection method, which is defined as finding the first disproportional increase of VE related to VO_2_ (6, 11, 12, 15). The POE represents the best integration between the respiratory and the cardiovascular system, because it corresponds to the moment during an incremental CPET at which there is the lowest ventilation to take up one litre of oxygen (9, 15).

But some authors have reported differences in the time-dependent sequence of AT and POE in less trained adults (6, 8, 16–18). While in some individuals POE and AT are found at the same work rate, for a similar number of individuals POE occurs at a lower work rate than AT indicating an earlier increase in VE in relation to VO_2_ (6). Therefore, it seems reasonable to distinguish between these two indices. Thus, the v-slope method for AT detection is the most direct approach to find the changes of the energy metabolism between phase one and phase two of the energy supply during incremental CPET. On the other hand, the POE reflects the ventilatory response of an individual to cope with the exercise strain during this initial phase of the CPET (2, 6). Therefore, training prescription based on POE could lead to training at other exercise intensities and, as a consequence, to different and unintended adaptions than training prescription based on AT (16).

In recent years the “Fatmax concept”, based on the crossover concept of fat and carbohydrate utilisation during exercise (19), has provided further ventilatory indices to describe changes concerning the fat metabolism due to exercise. Absolute fat oxidation rates increase during low to moderate exercise intensities until Fat_max_ (i.e., the exercise intensity, at which the maximal fat oxidation rate (MFO) occurs) and declines with a further increase in exercise intensity until it gets negligible, i.e., a minimum fat oxidation rate is reached (Fat_min_). Concomitantly, the carbohydrate (CHO) oxidation increases with the exercise intensity and becomes the dominant energy source at Fat_min_ and above (10, 20).

The fat metabolism is of great relevance in performance and health settings. Extensive use of fat oxidation during exercise reduces the requirement for endogenous carbohydrate oxidation and therefore muscle glycogen depletion, which is linked to fatigue. The capacity for fat oxidation during exercise has been associated with insulin sensitivity, weight gain, metabolic flexibility, and lower metabolic risk factors (20, 21). It has been shown that Fat_max_ occurs at 48%VO_2peak_ (22) or lower at 39%VO_2peak_ (23) in untrained individuals equalling exercise intensities below AT (22, 24). Additionally, Fat_max_ was reported to increase with training and enhancement of habitual physical activity (21, 23, 25, 26). On the other hand, Fat_max_ and AT were reported to occur at almost the same relative exercise intensity (45 vs. 46%VO_2peak_) in moderately trained men (27), indicating the interrelationship between Fat_max_ and AT. However, very large inter-individual variations and large discrepancies in the exercise intensity at Fat_max_ between specific population groups have been reported by various studies (10, 22, 28), which may be related to differences in the experimental design (different CPET-protocols; single- vs. multiple exercise tests) and the ventilatory indices detection methods (10, 21, 24). Therefore, the determination of Fat_max_ during a single incremental CPET and the analysis of the association to other ventilatory indices may be a valuable addition. Especially in clinical settings as an indicator of metabolic health and in performance settings where the capacity to utilize fat as a metabolic substrate is of concern (24).

While the influence of different determinants on single indices has already been studied extensively (2, 10), the sequencing patterns and the inter-relationship between the different ventilatory indices Fat_max_, POE and AT during a single CPET remain to be studied in order to understand differences between individuals (21, 24, 27).

In short, the time-dependent sequence of Fat_max_, POE and AT yield important insight into the metabolic and ventilatory response of individuals during low and moderate exercise intensities. Especially, the POE/AT-sequencing pattern may provide further information on different strategies to cope with the ventilatory and metabolic strain to exercise. It is, therefore, crucial to investigate the influence of determinants on the occurrence and the sequencing of these indices to further improve the assessment of the functional capacity of people to subsequently refine exercise-training prescription in less trained individuals.

Therefore, (1) the first aim of this study was to determine, whether different VISP groups concerning POE and AT exist in less trained adults. If distinct VISP groups were found, differences in participant characteristics, gas-exchange values and CPET results were presented. (2) The second aim was to investigate the association of the ventilatory index Fat_max_ with POE and AT.

## 2. Methods

### 2.1. Participants

**Figure 1:**
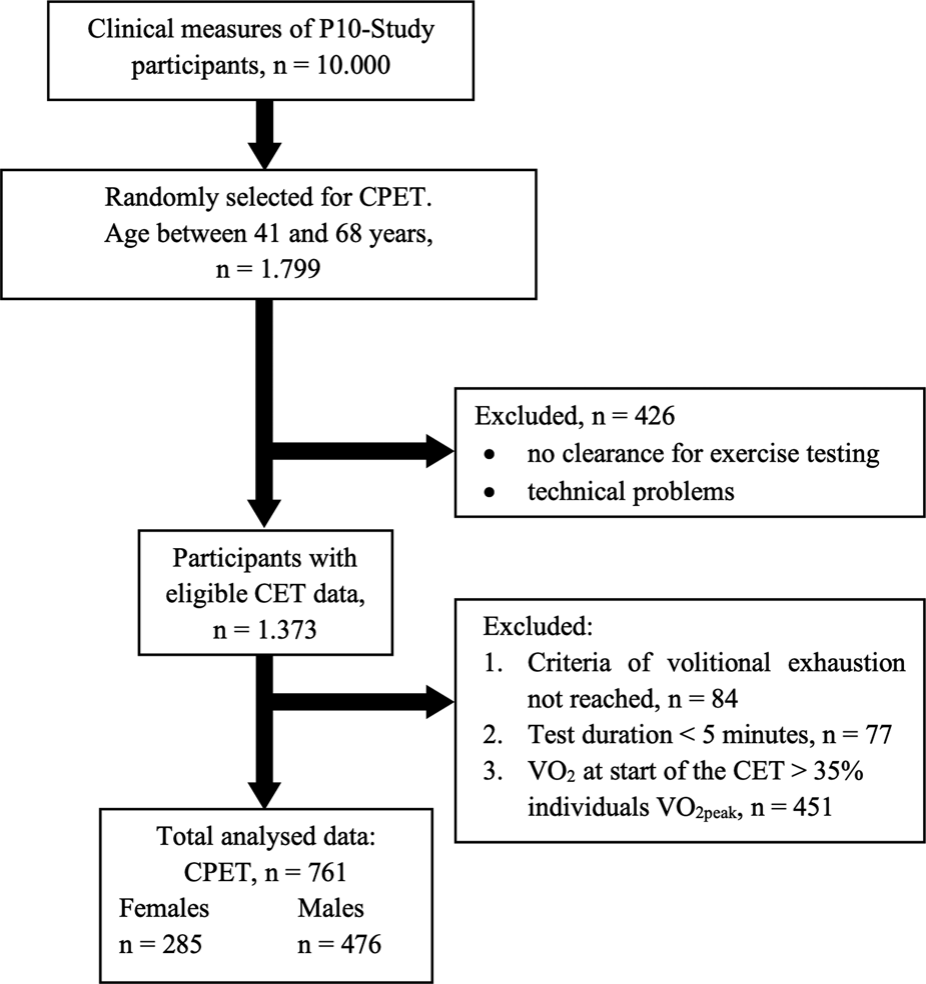
Cardiopulmonary exercise test (CPET) participant flow.

This is a cross-sectional study with 761 data-sets (285 females and 476 males) drawn from a sub-sample of 1.799 participants of the Paracelsus 10.000 Study (P10-Study) who were randomly assigned for CPET (Figure 1 and Table 2). The P10-Study was conducted between 2013 and 2020, and is a population based, observational study with the aim to investigate the state of health in 10.000 randomly selected 40 to 70 years old inhabitants of Salzburg, Austria (29). The P10-Study conformed to the principles outlined in the Declaration of Helsinki and was approved by the regional ethics committee of the federal state of Salzburg (415-E/1521/3–2012). All participants gave written informed consent.

### 2.2. Data collection

The measurements were supervised by the same investigators and were performed after overnight fasting at the Salzburg University Hospital, Austria between 7:00 a.m. and 3:00 *p*.m. The CPET measurements were performed between 1:00 and 3:00 *p*.m. Participants were instructed not to drink coffee or smoke on the test-day and were provided with standardized food. It consisted of a bread with cheese and water, consumed between 11:00 a.m. and noon.

#### 2.2.1. Participant characteristics and medical examinations

The medical examinations performed by physicians included a detailed medical history and physical examinations, anthropometric and standard gas-exchange measurements, laboratory evaluations (including blood chemistry, haematology and urine analysis), and an electrocardiogram. Body fat mass (FM) and fat free mass (FFM) were estimated by multi-frequency bio-impedance analysis (B.I.A Nutriguard-M, Data Input, Darmstadt, Germany). Therefore, electrodes (Bianostic AT, Data Input, Darmstadt, Germany) were attached on the frontal site of the left wrist and ankle of the participant lying in supine position and the measurements were made according to the manufacture's guidelines (Data Input, Darmstadt, Germany).

#### 2.2.2. CPET and gas exchange measurements

After the medical examination, participants were assigned for incremental exercise testing. Exclusion criteria for CPET were anaemia, cardiovascular disease, paralysis, abnormality of extremities, or other subjective limitations like pain or musculoskeletal disorders. During exercise, continuous respiratory gas analysis and volume measurements were performed using a facemask (Hans Rudolph, Kansas, United States) to ensure an airtight seal over the participant's nose and mouth with an attached volume sensor (Triple-V®) and a gas analyser (Master Screen CPX), which was connected using a semipermeable sampling tube (Twin Tube, all products are manufactured by Jaeger, Höchberg, Germany). The following parameters were recorded breath-by-breath throughout the exercise and registered as raw data: VO_2_, VCO_2_, VE, end-tidal partial pressure of oxygen and carbon dioxide (PETO_2_, PETCO_2_), ventilatory equivalents of O_2_ and CO_2_ (EQO_2_, EQCO_2_). Calibration of the equipment was performed every day by medical technicians according to the instruction manual using the inbuilt calibration tools and a reference gas (mixture of 5% CO_2_, 16% O_2_, 79% N_2_, Rießner Gase GmbH, Lichtenfels, Germany).

#### 2.2.3. CPET and exercise protocol

Each participant underwent an incremental exercise test until volitional exhaustion. The exercise protocols were designed to reach volitional exhaustion after 8 to 12 min of test duration (Table 1) using individual starting workloads and increments regarding sex and body mass-range as reported elsewhere (1, 30). The exercise test was performed on a cycle ergometer (ergo select 200P, ergo line GmbH, Bitz, Germany), and the height of the seat and the position of the handlebar was adjusted individually. After a 2 min stationary phase with no pedalling to allow the participants to become accustomed to breathing through the mask and a 2 min warm-up period at 10 W, an incremental exercise test with increasing workload every minute was performed until volitional exhaustion at a pedalling rate of 60 rpm. A 5-min recovery phase at 10 W was performed after exhaustion. Attainment of volitional exhaustion (and therefore VO_2peak_) was confirmed by at least two of the following criteria (3): (1) a plateau in VO_2_ (changes of less than 2 ml · kg^−1^ · min^−1^ following an increase in workload); (2) EQO_2_ > 30; (3) respiratory exchange ratio (RER) > 1.1; (4) achieving 90% of age predicted maximum heart rate (31); (5) pedalling rate < 50 rpm due to leg fatigue or shortness of breath. Exercise testing was terminated if any complications and contraindications occurred (1). During the incremental exercise test electrocardiogram was continuously recorded and blood pressure was determined every two minutes.

**Table 1:** Stationary cycling protocols of the P10-study for CPET.

	Females			Males			Females and Males
Body mass range, kg	50–69	50–69	70–94	50–69	70–94	70–94	95–119
Initial workload, W	40	50	60	50	70	70	90
Increment, W · min^−1^	10	10, 15^a^	10, 15^a^	15	15, 20^a^	20	20, 25^a^

^a^ increment rise after 6th minute to ensure a test duration of about 10 to 12 min.

### 2.3. Data processing

Data from the stationary cycling test (CPET) regarding warm-up and recovery phase were excluded from further analyses and the recorded breath-by-breath data were averaged over 10 s epochs. The mean of the three consecutive highest 10 s VO_2_ values at cessation was then taken as the peak value. Peak work rate (WR_peak_) was determined as the mean work rate during the last minute of the exercise test (32, 33).

POE and Fat_max_ are found at low to moderate exercise intensities and were reported at 44%–57%VO_2peak_ (8, 17) and 38%–64%VO_2peak_ (10, 21), respectively. Therefore, participants with a VO_2_ of more than 35% of the individual VO_2peak_ at the onset of stationary cycling were excluded from further analysis because the initial applied work rate might have been too high in these individuals to determine POE and Fat_max_. Furthermore, participants who could not complete at least five minutes of the CPET were excluded from further analysis.

The VO_2_ – work rate relationship (VO_2_/W slope) was assessed by linear regression of VO_2_ vs. work rate considering all exercise values up until VO_2peak_ (3).

#### 2.3.1. Determination of ventilatory indices

The ventilatory indices POE, AT and RCP were determined semiautomatic by combining automatic and visual detection methods (34). First, the indices were determined automatically using polynomial regression. Then, the time points of POE, AT und RCP during the exercise test were visually determined by finding the first disproportional increase in a VE (y-axis) vs. VO_2_ (x-axis) plot (9, 15), in a VCO_2_ vs. VO_2_ plot (14), and in a VE vs. VCO_2_ plot (3), respectively. In addition, EQCO_2_, PETCO_2_, EQO_2_, and PETO_2_ time plots were drawn as an additional guidance for the VI determination. The automatically detected indices were shown as a guide in these plots. Finally, the selected time points were then used to determine the work rate at POE, AT and RCP using the test protocol. The VO_2_ and VCO_2_ 10 s averages were used to calculate fat oxidation rates according to the non-protein respiratory exchange ratio (RER) technique with the assumption that the urinary nitrogen excretion rate was negligible (10). For each participant the calculated values for fat oxidation were depicted graphically as a function of exercise intensity (%VO_2 peak_) and a 3rd degree polynomial function with intersection in (0,0) was constructed to determine the relative intensity that elicited the highest rate of fat oxidation (Fat_max_) (10, 35). If less than six calculated fat oxidation values where available to construct the 3rd degree polynomial, the subject was excluded from further analysis (36).

#### 2.3.2. Ventilatory indices sequencing pattern (VISP) groups

To determine differences between the VISP-groups, the work rates at POE and at AT were determined and the investigated participants were categorized into the following VISP groups: (1) VISP_POE < AT_, participants with a lower work rate at POE compared to AT. (2) VISP_POE = AT_, participants without a difference in work rate between POE and AT.

### 2.4. Statistical analysis

Data are given as means ± standard deviation. Due to sex differences in energy metabolism and exercise performance, analysis were conducted separately for female and male participants. The Shapiro-Wilk test and visual inspection of histograms and quantile-quantile plots were used to verify the normal distribution of the data (*n* < 50). Differences in sex and in VISP groups for participant characteristics and CPET variables were tested using unpaired t-tests and Mann-Whitney non-parametric tests when appropriate. Mean biases [95% confidence intervals (CI)] ± 95% limits of agreement according to Bland and Altman were calculated to evaluate the level of absolute agreement between the ventilatory indices Fat_max_, POE and AT (37). The explained variance of the work rate differences between POE and AT and the work rate differences between Fat_max_ and AT on the aerobic capacity (measured as AT) were tested by linear regression analysis. Additionally, the relations between the relative oxygen uptake at Fat_max_ and the two ventilatory indices POE and AT were described by linear regression analysis using Pearson correlations.

A mixed-design ANOVA was completed to investigate differences between the ventilatory indices Fat_max_, POE and AT (within-subjects factor, VI) and between VISP groups (between-subjects factor, G) in relative oxygen uptake, relative work rates, relative heart rates and RER. Bonferroni *post-hoc* comparisons were applied when ANOVA indicated significant interaction effects. The level of significance was set at *α* ≤ .05. The statistical analyses were performed using RStudio version 1.2.5001 (RStudio Inc., Boston, Massachusetts, United States).

## 3. Results

### 3.1. Participant characteristics

Participant characteristics of the 285 females and 476 males who successfully completed the CPET and met the inclusion criteria for this study are listed in Tables 2, 3.

**Table 2:** Characteristics, comorbidity and main results of resting spirometry and cardiopulmonary exercise test (CPET) in females and males.

	Females		Males				
n	285		476				
	M	SD	M	SD	df	*t*	*p*
**Characteristics**							
Age, yrs	54	3	55	4	759	4.41	<.001
Body mass, kg	67	11	83	12	759	19.18	<.001
FM, kg	21	8	19	6	696	−3.66	<.001
Body fat, %	30	7	22	5	696	−17.18	<.001
FFM, kg	46	5	65	8	696	34.67	<.001
Height, m	1.7	0.1	1.8	0.1	720	27.57	<.001
BMI, kg · m^−2^	24.2	3.8	26.1	3.2	720	7.46	<.001
Waist circumference, cm	85	10	96	10	711	14.80	<.001
**Comorbidity**							
Hypertension, N (%)	22 (8)		75 (16)				
Pulmonary disease, N (%)	20 (7)		43 (9)				
Diabetes mellitus, N (%)	2 (1)		14 (4)				
Cardiovascular disease, N (%)	12 (4)		34 (8)				
**Resting Spirometry**							
FVC, L	3.6	0.6	4.9	0.7	488	19.98	<.001
FEV_1_, L	2.7	0.4	3.6	0.6	488	18.85	<.001
**CPET**							
Test duration, min	10.5	2.0	10.0	2.0	759	-3.53	<.001
%VO_2peak_ at start, %	30	4	28	4	759	-5.77	<.001
%VO_2peak_ at Fat_max_, %	41	8	43	7	728	3.64	<.001
%VO_2peak_ at POE, %	46	9	46	8	758	-0.57	.567
%VO_2peak_ at AT, %	53	9	54	9	759	0.35	.726
%VO_2peak_ at RCP, %	83	10	84	10	725	1.29	.197
VO_2peak_, ml · kg^−1^ · min^−1^	26.0	5.4	31.0	6.3	759	11.28	<.001
WR at start, W · kg^−1^	0.7	0.1	0.9	0.1	757	16.93	<.001
WR_peak_, W · kg^−1^	2.3	0.5	2.8	0.6	759	13.03	<.001
RER_peak_	1.19	0.07	1.20	0.08	759	2.22	.027
HR_peak_, min^−1^	164	12	163	13	741	-0.75	.451
VO_2_/WR slope, ml O_2_ ⋅ W^−1^	10.4	1.3	10.2	1.2	759	-1.85	.065

Data are presented as mean (M) ± standard deviation (SD) and frequencies (%); FM: fat mass; FFM: fat-free mass; BMI: body mass index; FVC: forced vital capacity; FEV_1_: forced expiratory volume over 1 s; WR: work rate; VO_2_: oxygen uptake; RER: respiratory exchange ratio; HR: heart rate; VO_2_/WR slope: VO_2_ – work rate relationship; *p* = significance level between males and females (unpaired *t*-tests).

**Table 3:** Characteristics, comorbidity and main results of resting spirometry and cardiopulmonary exercise test (CPET) in VISP_POE < AT_ and VISP_POE = AT_ participants, separately for females and males.

	VISP_POE < AT_		VISP_POE = AT_				
Females, *n*	197		88				
	M	SD	M	SD	df	t	p
**Characteristics**							
Age, yrs	54	3	54	3	283	1.32	.189
Body mass, kg	67	11	67	12	283	−0.47	.637
FM, kg	20	8	21	8	256	−0.55	.580
Body fat, %	30	6	30	7	256	−0.34	.736
FFM, kg	46	5	47	5	256	−0.26	.793
Height, m	1.7	0.1	1.7	0.1	269	0.27	.790
BMI, kg · m^−2^	24.1	3.7	24.4	4.0	269	−0.65	.515
Waist circumference, cm	84	10	87	11	266	−1.85	.065
**Comorbidity**							
Hypertension, N (%)	12 (6)		10 (12)				
Pulmonary disease, N (%)	17 (9)		3 (4)				
Diabetes mellitus, N (%)	1 (1)		1 (2)				
Cardiovascular disease, N (%)	6 (3)		6 (7)				
**Resting Spirometry**							
FVC, L	3.6	0.6	3.7	0.5	170	−0.70	.484
FEV_1_, L	2.7	0.4	2.8	0.5	170	−0.89	.372
**CPET**							
Test duration, min	10.7	2.0	10.0	2.1	283	2.84	.005
%VO_2peak_ at start, %	30	4	29	4	283	1.38	.169
%VO_2peak_ at RCP, %	84	10	81	11	274	1.83	.068
VO_2peak_, ml · kg^−1^ · min^−1^	26.3	5.2	25.5	5.7	283	1.08	.281
WR at start, W · kg^−1^	0.7	0.1	0.8	0.1	282	−1.14	.255
WR at RCP, W · kg^−1^	1.9	0.4	1.8	0.5	274	0.96	.338
WR_peak_, W · kg^−1^	2.3	0.4	2.2	0.5	283	1.64	.102
%HR_peak_ at RCP, %	90	6	90	6	269	−0.11	.913
HR_peak_, min^−1^	165	12	162	12	277	1.42	.158
RER at RCP	1.08	0.06	1.07	0.07	274	1.31	.191
RER_peak_	1.19	0.06	1.18	0.08	283	0.83	.410
VO_2_/WR slope, ml O_2_ ⋅ W^−1^	10.2	1.3	10.7	1.4	283	−2.67	.008

** **	VISP_POE < AT_		VISP_POE = AT_		** **	** **	** **
Males, *n*	307		169		** **	** **	** **
** **	M	SD	M	SD	df	t	p
**Characteristics**							
Age, yrs	55	4	55	4	474	−0.76	.450
Body mass, kg	84	12	83	11	474	0.23	.820
FM, kg	18	7	19	6	438	−1.11	.269
Body fat, %	22	6	23	5	438	−1.85	.065
FFM, kg	65	8	64	8	438	1.50	.135
Height, m	1.8	0.1	1.8	0.1	449	0.83	.405
BMI, kg · m^−2^	26.0	3.2	26.2	3.1	449	−0.56	.576
Waist circumference, cm	95	10	97	9	443	−1.38	.167
**Comorbidity**							
Hypertension, N (%)	42 (14)		33 (20)				
Pulmonary disease, N (%)	32 (11)		11 (7)				
Diabetes mellitus, N (%)	6 (3)		8 (7)				
Cardiovascular disease, N (%)	24 (8)		10 (6)				
**Resting Spirometry**							
FVC, L	4.8	0.7	4.9	0.7	316	−0.31	.755
FEV_1_, L	3.6	0.6	3.6	0.5	316	−0.38	.705
**CPET**							
Test duration, min	10.1	2.0	9.7	1.9	474	1.99	.047
%VO_2peak_ at start, %	28	4	28	4	474	−0.55	.584
%VO_2peak_ at RCP, %	85	10	82	11	449	2.11	.036
VO_2peak_, ml · kg^−1^ · min^−1^	31.4	6.6	30.3	5.6	474	1.85	.066
WR at start, W · kg^−1^	0.9	0.1	0.9	0.1	473	0.50	.619
WR at RCP, W · kg^−1^	2.3	0.6	2.2	0.6	449	2.15	.032
WR_peak_, W · kg^−1^	2.9	0.6	2.8	0.6	474	1.77	.077
%HR_peak_ at RCP, %	88	7	88	7	438	0.78	.435
HR_peak_, min^−1^	163	13	163	13	462	0.17	.863
RER at RCP	1.09	0.07	1.08	0.07	449	1.92	.055
RER_peak_	1.21	0.08	1.19	0.07	474	2.15	.032
VO_2_/WR slope, ml O_2_ ⋅ W^−1^	10.2	1.3	10.3	1.2	474	−0.92	.358

Data are presented as mean (M) ± standard deviation (SD) and frequencies (%); VISP: ventilatory indices sequencing pattern; FM: fat mass; FFM: fat-free mass; BMI: body mass index; FVC: forced vital capacity; FEV_1_: forced expiratory volume over 1 s; VO_2_: oxygen uptake; RCP: respiratory compensation point; WR: work rate; HR: heart rate; RER: respiratory exchange ratio; VO_2_/WR slope: VO_2_ – work rate relationship; *p* = significance level between the VISP groups (unpaired *t*-tests).

Compared to reference values published by Rapp et al. (2018), the female and male participants of this study are characterized by an average cardiorespiratory fitness corresponding approximately to the 50th and 40th percentile (in terms of VO_2peak_: 26.0 ± 5.4 vs. 31.0 ± 6.3 ml ⋅ kg^−1^ ⋅ min^−1^), respectively. VO_2peak_ ranged from 13.3 to 44.8 ml ⋅ kg^−1^ ⋅ min^−1^ in females and 16.2 to 55.5 ml ⋅ kg^−1^ ⋅ min^−1^ in males. The females and males displayed normal pulmonary function at rest with an average FVC of 3.6 ± 0.6 l and 4.9 ± 0.7 l, and an average FEV_1_ of 2.7 ± 0.4 l and 3.6 ± 0.6 l, respectively (38). The female participants in this study were marginally younger, were on average 16 kg lighter and had 11 cm lower waist circumference than the males. Additionally, females possessed a higher FM in kg as well as a higher percentage of body fat than males but a lower FVC and FEV_1_.

Furthermore, there was a sex difference in VO_2peak_, WR_peak_, and %VO_2peak_ at Fat_max_ being significantly higher in males. But no significant sex differences were found at the remaining ventilatory indices POE, AT, and RCP (in terms of %VO_2peak_). Regarding sex differences in the test protocol, we found a significant higher starting work rate in males, and a longer test duration and higher %VO_2peak_ at test start in females.

### 3.2. Differences in work rates between POE and AT

Based on a difference in work rates between POE and AT, participants were divided into two VISP groups (Tables 3, 4). Thus, 197 females and 307 males who showed a difference in work rate between POE and AT were assigned to VISP_POE < AT_. This corresponds to 66% of all study participants. The remaining 88 females and 169 males with no difference were assigned to VISP_POE = AT_ (Tables 3, 4). The intra-group distribution (VISP_POE < AT_ vs. VISP_POE = AT_) was similar between females (69% vs. 31%) and males (64% vs. 36%). The female and male characteristics, gas-exchange values and CPET results in VISP_POE < AT_ and VISP_POE = AT_ participants differed in test duration (females: 10.7 ± 2.0 vs. 10.0 ± 2.1 min; males: 10.7 ± 2.0 vs. 10.0 ± 2.1 min), relative oxygen uptake and relative work rate at RCP (males: 85 ± 10 vs. 82 ± 11%VO_2peak_; 2.3 ± 0.6 vs. 2.2 ± 0.6 W · kg^−1^), VO_2_ – work rate relationship (females: 10.2 ± 1.3 vs. 10.7 ± 1.4 ml O_2_ ⋅ W^−1^) and RER_peak_ (males: 1.21 ± 0.08 vs. 1.19 ± 0.07) (Table 3).

**Table 4:** Relative oxygen uptake, absolute and relative work rate, relative heart rate and respiratory exchange ratio at the ventilatory indices Fat_max_, POE and AT in VISP_POE < AT_ and VISP_POE = AT_ participants.

Females, *n*	VISP_POE < AT_		VISP_POE = AT_		ANOVA			
	197		88					
	M	SD	M	SD	Effect	*F* ratio	df	*η*_p_^2^
**Relative oxygen uptake, %VO_2peak_**								
Fat_max_	42	8	41	8	G	0.35	1	.00
POE	45	8	49	9	VI	239.60***	2	.47
AT	55	9	50	8	G × VI	38.80***	2	.13
**Absolute work rate, W**								
Fat_max_	55.6	12.0	56.5	12.2	G	0.24	1	.00
POE	59.3	14.7	68.2	14.3	VI	156.70***	2	.37
AT	75.3	18.7	68.0	14.1	G × VI	42.90***	2	.14
**Relative work rate, W · kg^−1^**								
Fat_max_	0.9	0.2	0.8	0.2	G	0.01	1	.00
POE	0.9	0.2	1.0	0.2	VI	153.60***	2	.36
AT	1.2	0.3	1.0	0.2	G × VI	41.50***	2	.13
**Relative heart rate, %HR_peak_**								
Fat_max_	64	6	65	6	G	2.05	1	.01
POE	66	7	70	7	VI	189.80***	2	.42
AT	72	8	70	6	G × VI	30.7***	2	.10
**Respiratory Exchange Ratio**								
Fat_max_	0.85	0.05	0.84	0.07	G	2.40	1	.01
POE	0.86	0.06	0.87	0.08	VI	192.00***	2	.41
AT	0.92	0.07	0.88	0.08	G × VI	38.80***	2	.13

Males, *n*	VISP_POE < AT_		VISP_POE = AT_		ANOVA			
	307		169					
	M	SD	M	SD	Effect	*F* ratio	df	η_p_^2^
**Relative oxygen uptake, %VO_2peak_**								
Fat_max_	44	7	43	7	G	0.54	1	.00
POE	43	7	50	8	VI	346.80***	2	.43
AT	55	9	51	8	G × VI	99.70***	2	.18
**Absolute work rate, W**								
Fat_max_	89.1	17.6	87.1	19.2	G	0.06	1	.00
POE	86.5	17.3	102.0	26.8	VI	234.00***	2	.34
AT	115.0	27.1	102.0	26.3	G × VI	111.00***	2	.20
**Relative work rate, W · kg^−1^**								
Fat_max_	1.1	0.2	1.1	0.3	G	0.04	1	.00
POE	1.1	0.2	1.2	0.4	VI	219.00***	2	.33
AT	1.4	0.4	1.2	0.3	G × VI	103.00***	2	.19
**Relative heart rate, %HR_peak_**								
Fat_max_	64	6	64	6	G	1.75	1	.00
POE	64	7	67	7	VI	249.50***	2	.36
AT	70	7	68	7	G × VI	72.50***	2	.14
**Respiratory Exchange Ratio**								
Fat_max_	0.85	0.05	0.85	0.05	G	0.62	1	.00
POE	0.86	0.06	0.89	0.07	VI	366.00***	2	.45
AT	0.92	0.07	0.89	0.07	G × VI	100.00***	2	.18

Data are presented as mean (M) ± standard deviation (SD); ANOVA = analysis of variance; G = group (VISP_POE < AT_, VISP_POE = AT_); VI = ventilatory indices (Fat_max_, POE and AT).

*** *p* < .001.

### 3.3. Associations between the ventilatory indices Fat_max_, POE and AT

**Figure 2:**
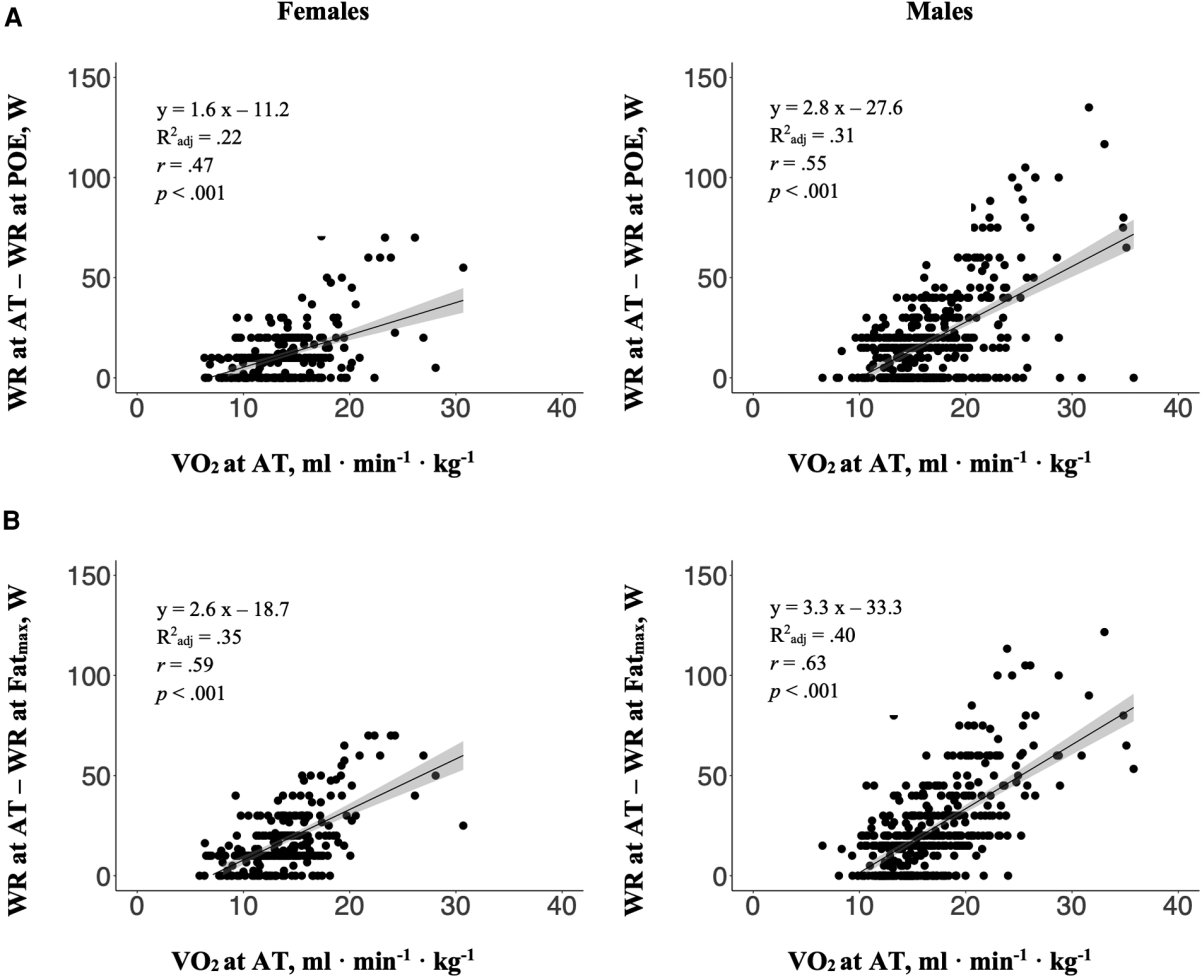
The relations between the aerobic capacity (in terms of VO_2_ at AT) and the magnitude of the work rate (WR) differences (**A**) between POE and AT, and (**B**) between Fat_max_ and AT, separately for females and males. Dark grey area: 95% confidence interval; R^2^_adj_: Adjusted R-square; r: Pearson's product-moment correlation coefficient.

The mean (± standard deviation) work rate difference between POE and AT of VISP_POE < AT_ participants was significantly higher in males than in females (28 ± 21 W vs. 17 ± 12 W). There was a significant correlation between the aerobic capacity (in terms of VO_2_ at AT) and the magnitude of the work rate differences between POE and AT, with *r* = .47 (females: *p* < .001) and *r* = .55 (males: *p* < .001) (39) and with coefficient of determination (R^2^_adj_) explaining 22% and 31% of the variance in work rate difference of females and males, respectively (Figure 2A). Additionally, there was a significant correlation between the aerobic capacity and the magnitude of work rate difference between Fat_max_ and AT, with *r* = .59 (females: *p* < .001) and *r* = .63 (males: *p* < .001) (39) and with coefficient of determination (R^2^_adj_) explaining 35% and 40% of the variance in work rate difference of females and males, respectively (Figure 2B).

**Figure 3:**
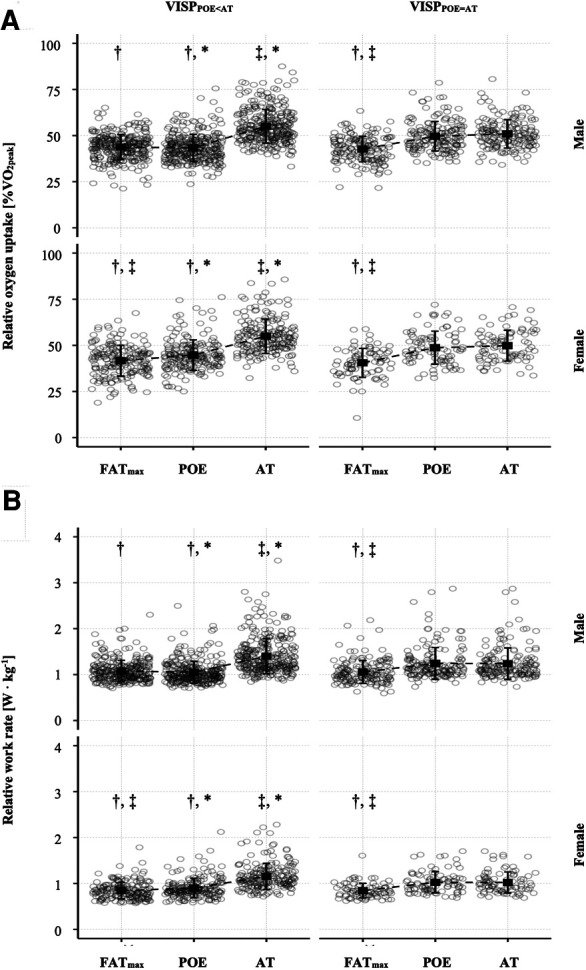
Means (squares) ± standard deviations (lines) of the relative oxygen uptake (**A**) and the relative work rate (**B**) at the ventilatory indices Fat_max_, POE and AT in VISP_POE < AT_ and VISP_POE = AT_, shown for females and males separately. The dots refer to individual values at the different ventilatory indices. Additionally, adjusted *p*-values of *post-hoc* comparisons between ventilatory indices and VISP groups are displayed: ^†^*p* < .05 vs. AT; ^‡^*p* < .05 vs. POE; *p < .05 vs. VISP_POE = AT_.

There was a significant interaction between the ventilatory indices (Fat_max_, POE and AT) and VISP groups (VISP_POE < AT_ and VISP_POE = AT_) for the relative oxygen uptake (in terms of %VO_2peak_), the relative work rate (in terms of W · kg^−1^), the relative heart rate (in terms of %HR_peak_) and the respiratory exchange ratio at the different ventilatory indices in females as well as in males (Table 4). *Post-hoc* pairwise comparisons indicated significant differences between VISP_POE < AT_ and VISP_POE = AT_ participants (Figure 3). The relative oxygen uptake and the relative work rate at POE were significantly lower in VISP_POE < AT_ compared to VISP_POE = AT_ participants, while both were significantly higher at the AT in VISP_POE < AT_ compared to VISP_POE = AT_ participants. Additionally, AT was significantly higher compared to Fat_max_ in both groups.

We used a Bland-Altman limit of agreement analysis to investigate the absolute agreement between the ventilatory indices Fat_max_, POE and AT. In VISP_POE < AT_ participants, Fat_max_ was best associated with POE (mean bias [95% CI] ranging from −3 [−4; −2] to 0 [−1;1] %VO_2peak_). The agreement with AT was lower (mean bias [95% CI] ranging from −13 [−14; −12] to −11 [−12, −10] %VO_2peak_). In VISP_POE = AT_ participants, there was a similar agreement of Fat_max_ with both, the POE and AT. As expected, there was a high agreement between POE and AT in VISP_POE = AT_ participants (mean bias [95% CI] of −1 [−2; 0] and −1 [−2; −1] %VO_2peak_) and a low agreement in VISP_POE < AT_ participants (mean bias [95% CI] of −10 [−11; −9] and −12 [-13; −11] %VO_2peak_). Results were similar in females and males (Table 5).

**Table 5:** Bland-Altman analysis. Comparison of the relative oxygen uptake (in terms of %VO2_peak_) between the ventilatory indices Fat_max_, POE and AT in VISP_POE < AT_ and VISP_POE = AT_ participants, separately for females and males.

	Females		Males	
	VISP_POE < AT_	VISP_POE = AT_	VISP_POE < AT_	VISP_POE = AT_
Fat_max_ vs. POE	−3 [−4; −2] ± 17	−8 [−10; −7] ± 17	0 [−1; 1] ± 15	−7 [−8; −6] ± 16
Fat_max_ vs. AT	−13 [−14; −12] ± 18	−9 [−11; −8] ± 16	−11 [−12; −10] ± 18	−8 [−10; −7] ± 15
POE vs. AT	−10 [−11; −9] ± 14	−1 [−2; 0] ± 06	−12 [−13; −11] ± 15	−1 [−2; −1] ± 05

Data are presented as mean bias [95% confidence interval] ± 95% limits of agreement.

**Figure 4:**
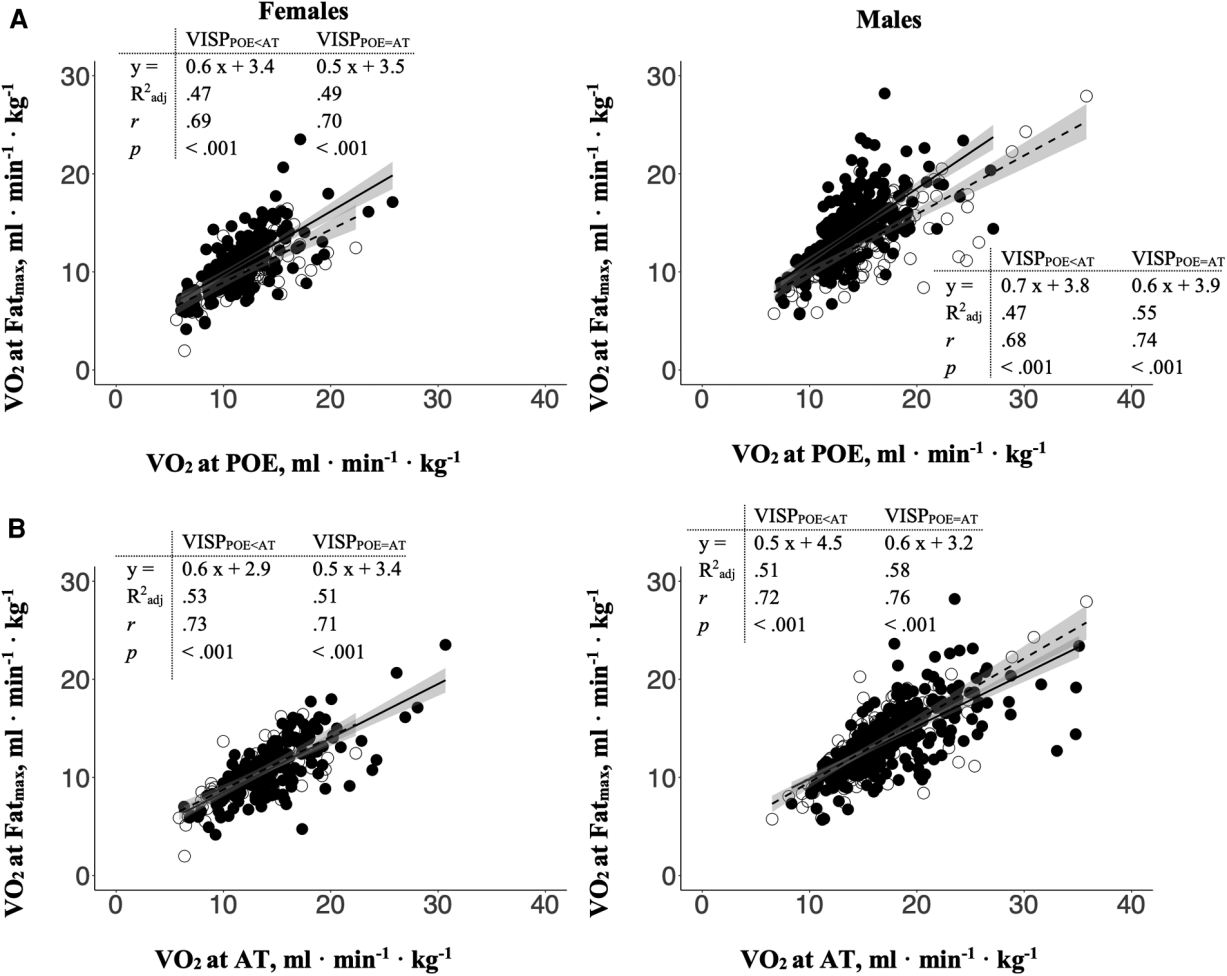
The relations between the relative oxygen uptake at Fat_max_ with (**A**) the relative oxygen uptake at POE and (**B**) AT in VISP_POE < AT_ (closed circles and solid line) and VISP_POE = AT_ (open circles and dashed line), separately for females and males. Dark grey area: 95% confidence interval; R^2^_adj_: Adjusted R-square; r: Pearson's product-moment correlation coefficient.

There was a strong correlation between the relative oxygen uptake at Fat_max_ and the two ventilatory indices POE and AT, with *r* ranging from.68 to.76 (*p* < .001) (39) and with coefficient of determination (R^2^_adj_) explaining 47% to 58% of the variance in VO_2_ at Fat_max_. No VISP group differences could be discerned (Figure 4).

## 4. Discussion

Ventilatory indices are valuable indicators to assess the metabolic and ventilatory response during exercise in individuals. Combining different indices into a single CPET may yield great potential to further improve the interpretation of CPET results. Therefore, we determined whether different groups of sequencing patterns in ventilatory indices (VISP) concerning POE and AT in less trained females and males exist. Further, we analysed the fat metabolism (Fat_max_) during CPET to support the hypothesis, that differences between VISP groups are to some extent explained by inter-individual differences in the ability to oxidize fat during exercise.

### 4.1. Participant characteristics and sex differences

As expected, the females in this study presented with a lower cardiorespiratory fitness in terms of VO_2peak_ and a lower pulmonary function (FEV_1_ and FVC) than the males (10, 38, 40). Additionally, the females possessed significant lower absolute work rates and oxygen uptake levels at the ventilatory indices Fat_max_, POE and AT. This is in line with data reported by others and is related to differences in body mass and composition, lung size and a greater reliance on fat metabolism during exercise in females (21, 38). Interestingly, when work rate and oxygen uptake were expressed in relation to the peak values, no sex differences could be found. Only a marginal lower Fat_max_ (41 ± 8 vs. 43 ± 7%VO_2peak_) was found in females compared to males (Table 2). This is in contrast to others reporting a minor tendency towards a greater Fat_max_ in females compared to males (56 ± 14 vs. 51 ± 14%VO_2peak_) (21). It has been shown that there is a large inter-individual variation in fat oxidation with physical activity, VO_2peak_ and sex only explaining 12% of the inter-individual variation in MFO. Hence, genetic predisposition, diet or menopausal mechanisms may further contribute to variations in fat oxidation (22, 41). Nevertheless, it seems reasonable to analyse females and males separately when differences in fat metabolism during exercise are of interest.

### 4.2. Differences in work rates between POE and AT

Based on the work rate differences between POE and AT, we identified two VISP groups: 66% of the total sample presented with a significant lower work rate at POE compared to AT (VISP_POE < AT_), while the remaining participants showed no POE - AT work rate difference (VISP_POE = AT_). An early occurrence of POE compared to AT has also been reported by others (8, 17, 18). We calculated a mean work rate difference between POE and AT of 28 ± 21 W in females and 17 ± 12 W in males of the VISP_POE < AT_ group, corresponding to 12% and 11% of WR_peak_, respectively. These differences cannot be neglected when CPET is used to evaluate and prescribe exercise training intensity or to assess the functional capacity of individuals.

Traditionally, POE and AT are considered the same ventilatory indices and are used interchangeable to detect the transition between phase one and phase two of the three-phase model of energy supply (2, 11, 12). AT is determined by identifying the first disproportional increase in VCO_2_ related to VO_2_, resulting from the “excess CO_2_” due to the additional allocation of ATP by anaerobic glycolysis as the exercise intensity increases (14). This “excess CO_2_” must be eliminated *via* enhanced ventilation and consequently, there will be a disproportional increase in VE related to VO_2_, which corresponds to the POE (3, 12). But some factors seem to disrupt this close link between VE and VCO_2_ in VISP_POE < AT_ participants of this study, as indicated by the early increase in VE (at POE) followed by the subsequent increase in VCO_2_ at a higher work rate (at AT).

In a recent study (6) we tried to identify these factors and therefore, analysed breathing pattern differences by breathing frequency and tidal volume. But we could not find any interrelationships to explain the early increase in VE independent of the later “excess CO_2_”. Accordingly, we concluded in agreement with others that the VE vs. VO_2_ relation (POE) is not suitable for determining the AT (5). Because of that observation, the POE should be seen as a distinct ventilatory index, describing the ventilatory adaptions and strategies of an individual in response to increasing exercise intensity (6, 8).

The participants of VISP_POE < AT_ and VISP_POE = AT_ are comparable in regard to age, anthropometric characteristics, resting spirometric values and peak CPET results. Additionally, VISP_POE < AT_ and VISP_POE = AT_ participants started the exercise test at comparable work rates and oxygen uptake levels (Table 3). Therefore, an influence of the test protocol can be excluded.

Further, no differences between VISP_POE < AT_ and VISP_POE = AT_ participants were found at Fat_max_. Interestingly, while there was no difference in the aerobic power (VO_2peak_) we found a significant higher aerobic capacity (in terms of %VO_2peak_ at AT) and a significant higher relative work rate at AT (in terms of W· kg^−1^) in VISP_POE < AT_ compared to VISP_POE = AT_ participants Additionally, the first disproportional increase in VE at POE occurred at a significant lower relative oxygen uptake and a significant lower relative work rate in VISP_POE < AT_ compared to VISP_POE = AT_ participants (Figure 3). Therefore, an early increase in ventilation, indicated by the POE at a lower work rate compared to AT, may lead to a delay in the appearance of the AT and can be seen as a ventilatory strategy adopted by some individuals (VISP_POE < AT_).

There was a significantly lower VO_2_ – work rate relationship in VISP_POE < AT_ compared to VISP_POE = AT_ females (10.2 ± 1.3 vs. 10.7 ± 1.4 ml O_2_ ⋅ W^−1^) but only a tendency towards a lower VO_2_ – work rate relationship in VISP_POE < AT_ males (10.2 ± 1.3 vs. 10.3 ± 1.2 ml O_2_ ⋅ W^−1^). A flattening of the VO_2_ – work rate relationship has been related to decreased work of breathing (3) and to improvements in the locomotor-respiratory coupling, associated with a decrease of the metabolic requirements during exercise by reducing respiratory muscle fatigue (42). Therefore, an early increase in ventilation (POE at a lower work rate compared to AT) may therefore be another ventilatory adaption to regular exercise and decrease the work of breathing.

### 4.3. Associations between the ventilatory indices Fat_max_, POE and AT

POE and AT were found at 46 ± 8 and 54 ± 9%VO_2peak_ (1.1 ± 0.3 and 1.3 ± 0.4 W ⋅ kg^−1^) in the total sample, which is in agreement with other studies (2, 8, 17). The POE reflects the best integration of ventilation and the cardiorespiratory system with the most efficient ventilation in terms of O_2_ extraction. It means that this is the point during an exercise test, where the least ventilation is required for the uptake of one litre of oxygen (8, 9). In a recent study by Ramos et al. (2012), POE was reported to occur at 44%VO_2peak_ (range: 30%–50%VO_2peak_) in 2,237 untrained adults, always present at lower exercise intensities compared to AT. In our study, this result is in line with participants of the VISP_POE < AT_ but not the VISP_POE = AT_ group, shown by the Bland-Altman analysis (Table 5). Slightly different methods of POE determination (Ramos et al. (8) detected POE by determining the lowest value of the ratio between VE and VO_2_) may explain these differences.

The ventilatory index Fat_max_ was found at a significant lower exercise intensity (in terms of %VO_2peak_ and W · kg^−1^) compared to AT in both groups (Figure 3). This is in line with other studies, reporting Fat_max_ at lower exercise intensities compared to AT in untrained adults (22, 24). In contrast, similar values for Fat_max_ and AT (45 ± 8 vs. 47 ± 10%VO_2peak_) were found in trained male cyclists (27). An increase in Fat_max_ was reported after endurance training, but only in previously sedentary individuals (26). In previously physically active or trained individuals, only a small tendency towards a Fat_max_ increase was reported (21). On the other hand, the submaximal fat oxidation rates (e.g., MFO) can largely increase with training in sedentary as well as physically active and trained individuals (10, 21, 26). In conclusion, it seems that the difference between Fat_max_ and AT decrease with endurance training because of increased full-body fat oxidation caused by skeletal muscle adaptions (e.g., mitochondrial biogenesis, increased tricarboxylic acid cycle enzyme and electron transport chain protein content, and increased fatty acid transporter and enzyme content (21)) leading to an increased Fat_max_ and MFO.

In our study, there was a strong positive linear relationship in VISP_POE < AT_ and VISP_POE = AT_ females and males (ranging from *r* = .71 to *r* = .76) between Fat_max_ and the aerobic capacity (in terms of %VO_2peak_ at AT) confirming the association of Fat_max_ and training status. Additionally, we found a strong positive linear relationship (*r* = .59 in females and *r* = .63 in males) between the aerobic capacity and the work rate difference between AT and Fat_max_, but with the coefficient of determination (R^2^_adj_) only explaining 35% and 40% (in females and males, respectively) of the variance in the aerobic capacity (Figure 2B). This contradicts the hypothesis stated above suggesting that the difference between Fat_max_ and AT decreases with an increase in aerobic capacity (e.g., as a consequence of endurance training). However, this could be related to the large inter-individual variations in this study, which diminish this effect. Specific experimental studies are needed to analyse this phenomenon.

No differences in Fat_max_ were found between VISP_POE < AT_ and VISP_POE = AT_ participants, and Fat_max_ was significantly lower than AT (in terms of %VO_2peak_ and W ⋅ kg^−1^) in both VISP groups. Interestingly, there was a strong agreement between Fat_max_ and POE in VISP_POE < AT_ (mean bias [95% CI] ranging from −3 [-4; −2] to 0 [-1; 1] %VO_2peak_), but not in VISP_POE = AT_ participants. This can be explained by the significant lower POE (in terms of %VO_2peak_ and relative work rate) in VISP_POE < AT_ compared to VISP_POE = AT_ participants. Consequently, differences in Fat_max_ between VISP_POE < AT_ and VISP_POE = AT_ participants do not seem to explain the work rate difference between POE and AT.

There is a strong inverse relationship between fat oxidation and blood lactate, and there is a major effect of lactatemia in limiting fat oxidation in individuals with widely ranging exercise capacities. Lactatemia significantly affects and downregulates fat metabolism with increasing exercise intensity. It has been shown, that physically active and trained individuals have a higher capacity to oxidize fat compared to sedentary individuals or individuals with mitochondrial dysfunction as a result of type-2-diabetes-mellitus or the metabolic syndrome (20). In this study we confirmed these findings by showing a strong correlation between Fat_max_ and the aerobic capacity (Figure 4). However, great inter-individual variation in Fat_max_ must be kept in mind. Beside sex, comorbidities (e.g., hypertension and diabetes mellitus) and training status, the menopause status and diet are reported to influence Fat_max_. Although, participants were instructed to realize overnight fasting and were provided with standardized food on the test day, the chronic nutritional status and the menstrual cycle of the female participants were not controlled. Hence, these factors should be taken into account in future studies when Fat_max_ is analysed and should be considered when interpreting the results of this study (22, 41).

Furthermore, the optimal test stage duration to determine submaximal and peak ventilatory indices in one single CPET does not exist. It has been shown, that MFO is slightly overestimated using a one minute incremental protocol, but Fat_max_ intensity (in terms of %VO_2peak_) is not affected by step duration (36). There is a delay in the increase of VO_2_ in response to increasing exercise intensity, which is known as the mean response time and which increases with exercise intensity and is dependent on the work rate increase per minute (43). Additionally, dependent on the fitness level of the participants, suitable stage durations may vary between individuals. But it has been shown that short time test protocols can be used to estimate different ventilatory indices in one single CPET (27, 36). Hence, we used different 1-minute stage-exercise protocols and increased the increment in some of our test protocols after the 6th minute of the test (Table 1) to realise, (1) a slow increase of the work rate during the early stages of the exercise test in order to minimise the VO_2_ response time, and (2) to be able to achieve reliable VO_2peak_ values by realising the recommended test duration of 8 to 12 min (30). The mean VO_2_ – work rate relationship in this study was found to be normal (10.3 ± 1.3 ml O_2_ · W^−1^) (1) but marked inter-individual variations were found (range: 5.5–17.5 ml O_2_ · W^−1^). These differences must be considered when interpreting the results of this study and may explain the great inter-individual variations in Fat_max_. Furthermore, the differences between the ventilatory indices POE and AT can be explained, at least partly, by the measurement error in determining the individual ventilatory indices. Consequently, further studies are needed to establish reliable test protocols and reference values for different study populations in order to analyse VISP using one single CPET (27).

### 4.4. Conclusion

In summary, there are differences in the time-dependent sequence of the ventilatory indices POE and AT in less trained adults. In some individuals the POE occurs at a lower work rate compared to AT (VISP_POE < AT_), while in the remaining individuals POE equals AT (VISP_POE = AT_). Therefore, POE should not be used to determine AT. Training prescription based on POE may result in different exercise intensity recommendations compared to AT and therefore, lead to different and unintended physiological adaptions.

Differences in Fat_max_ have not been found to influence the POE – AT work rate differences. Although participants with a POE – AT work rate difference were characterized by a higher aerobic capacity (in terms of %VO_2peak_ and W · kg^−1^ at AT), compared to participants with a work rate at POE equalling AT.

Therefore, it seems reasonable that the POE – AT work rate difference is mainly influenced by differences in the ventilatory response to the exercise strain at the early stages of the exercise test rather than by metabolic differences. And consequently, this ventilatory response leads to an increased aerobic capacity (in terms of %VO_2peak_) in those individuals.

Finally, we consider the determination of different ventilatory indices in a single CPET useful for the evaluation and interpretation of the ventilatory and metabolic response to exercise to provide a more comprehensive picture of the performance capacity of the tested individual. Additionally, the inclusion of further indices like the crossover point (19, 24) or the mitochondrial threshold (44) may add further value to our understanding of cardiorespiratory fitness measured by CPET. Yet, because of the complexity of the CPET results (e.g., sequencing patterns), advanced methods like neural networks (45) are underway to support a more comprehensive interpretation of the exercise testing from the very sedentary to the highly performative individual.

## Data Availability

The raw data supporting the conclusions of this article will be made available by the authors, without undue reservation.
